# Pathways to Fatherhood: Psychological Well-Being Among Israeli Gay Fathers Through Surrogacy, Gay Fathers Through Previous Heterosexual Relationships, and Heterosexual Fathers

**DOI:** 10.3389/fpsyg.2020.00091

**Published:** 2020-01-29

**Authors:** Geva Shenkman, Ofer Siboni, Fiona Tasker, Pedro Alexandre Costa

**Affiliations:** ^1^School of Psychology, Interdisciplinary Center Herzliya, Herzliya, Israel; ^2^Department of Psychological Sciences, Birkbeck, University of London, London, United Kingdom; ^3^William James Center for Research, ISPA – Instituto Universitário, Lisbon, Portugal

**Keywords:** gay fathers, same-sex parenting, surrogacy, parenthood satisfaction, Big Five, personality dimensions, sexual orientation, well-being

## Abstract

This study explored differences in psychological well-being as assessed by life satisfaction, parenthood satisfaction, depressive symptoms and the Big Five personality dimensions among 219 Israeli fathers; 76 gay men who had become fathers through a heterosexual relationship, 63 gay men who had become fathers through surrogacy, and 78 heterosexual men. After controlling for sociodemographic characteristics, gay fathers through surrogacy reported greater satisfaction with parenthood, greater satisfaction with their lives, and reported higher levels of extraversion when compared to heterosexual fathers. No significant differences emerged between the three groups on depressive symptoms, neuroticism, conscientiousness, agreeableness, and openness to experience. These findings emphasize the predominant similarities and some possible differences on psychological well-being between the different paths to fatherhood. This study is one of the first to compare several paths to fatherhood on psychological well-being, thus illuminating the contribution of fatherhood route to psychological well-being in an era where gay men are increasingly becoming fathers in diverse ways.

## Introduction

In light of social, political, and technological developments, gay men are becoming fathers nowadays more than ever before (Carneiro et al., 2017; Carone et al., 2017b). Gay fatherhood has attracted growing research attention in recent years in varied countries (e.g., Tornello et al., 2011; Shenkman and Shmotkin, 2014; Baiocco et al., 2018; Bos et al., 2018), and has focused both on the development of children of gay fathers, alongside the psychological functioning of the parents themselves (e.g., Goldberg et al., 2010; Tornello and Patterson, 2015; Tornello et al., 2015; Shenkman and Shmotkin, 2016, 2019; Farr, 2017; Patterson, 2017; Green et al., 2019), yet little attention has been given to the comparison between different paths to gay fatherhood both in the developmental domain of the children and the psychological well-being of the parents (Tasker, 2013). Thus, this study aims to examine the broad concept of psychological well-being (as indicated by parenthood satisfaction, depressive symptoms, life satisfaction, and the Big Five personality dimensions) among three groups of Israeli fathers: gay men who had become fathers through surrogacy, gay fathers through a previous heterosexual relationship, and heterosexual fathers.

Our current study dwells in the theoretical framework of the family systems theory (Cox and Paley, 1997) which suggests that the development and adaptation of both children and parents are influenced and shaped not only by the family subsystems (e.g., parents and children) but also by the broader socio-cultural context. The sociocultural environment of Israel is a particularly rich terrain for exploring the similarities and differences in psychological well-being as a function of fatherhood route. On the one hand, a familistic society, which Israel is a prime example, promotes and values childbearing more highly than many other Western nations and sanctifies parenthood as the primary path to social acceptance (Tsfati and Ben-Ari, 2019). On the other hand, Israel enacts multiple legal hardships upon gay men who wish to become parents. For example, surrogacy services are not legal for same-sex couples in Israel though they are legal for heterosexual couples, and gay men who wish to become parents via surrogacy turn to highly expensive overseas surrogacy services in South East Asia and North America (Teman, 2010). In addition, the opportunities for gay men to adopt are extremely restricted (Gross, 2014), which makes the surrogacy path, though encompassing multiple ordeals for Israeli gay men, one of the preferred routs to gay fatherhood (Birenbaum-Carmeli, 2016; Tsfati and Ben-Ari, 2019). Succeeding to achieve fatherhood through this desired but difficult path might be linked with a gain in well-being among gay men (e.g., Erez and Shenkman, 2016) and therefore, stands as one of the primary rationales for expecting differences in psychological well-being outcomes as a function of fatherhood route. Also, the above-mentioned conflicting messages from the Israeli socio-cultural context, make it even more interesting to understand whether the different paths chosen by gay fathers relate simply to cohort differences or psychological characteristics, or whether these pathways are linked to distinct differences in psychological well-being in comparison to the patterns recorded by heterosexual fathers.

The current study adopted a comparative approach to examine differences between the three paths to fatherhood. This comparative approach in the context of LGBT families has previously produced important information regarding disparities between heterosexual and gay/lesbian parents with respect to marital and parental rights, division of labor, and well-being (e.g., Hatzenbuehler et al., 2010; Reczek and Umberson, 2012; Shenkman, 2018). However, the comparative approach between gay/lesbian and heterosexual parented families also has been criticized as between−group designs focus primarily on differences based on sexual identities, while other identities that are salient to the experience of LGBT individuals and families become invisible (Fish and Russell, 2018). Our current comparative design, included both a comparison with heterosexual fathers and a comparison between two pathways and experiences of gay fatherhood. Thus, we cast light on different experiences of gay fatherhood while also keeping a point of comparison with heterosexual counterparts.

### Pathways to Gay Fatherhood

Four common routes are associated with gay fatherhood worldwide (e.g., Tasker and Patterson, 2007). These include gay men who had become fathers through a previous heterosexual relationship; gay men who had become fathers through adoption; gay men who had become fathers through shared parenting in agreement with a woman; and gay men who had become fathers through surrogacy. In the current study we will focus on the first and last, which are commonly considered as the most distinct in representing two polar social contexts (Tornello and Patterson, 2015). Gay parenting through a previous heterosexual relationship is commonly associated with fatherhood among middle-aged and older gay men who grew up in an environment in which their sexual orientation was considered as pathological and opportunities to become a parent outside of a heterosexual relationship were almost non-existent (Morrow, 2001; Tasker and Patterson, 2007; Tasker, 2013). In contrast gay fatherhood through surrogacy is associated with contemporary planned gay-fathers families, and is achieved through the use of progressive fertility technologies involving donated eggs, *in vitro* fertilization, and surrogacy with at least some liberal state policies allowing gay men access to these procedures (e.g., Carone et al., 2018a, b). Extremely scarce are studies that directly compare between different pathways to gay fatherhood (e.g., Carroll, 2018), probably because of difficulties inherent in achieving sufficient sample size for each group to allow quantitative comparisons (Roy et al., 2015). Thus, many studies that focus on gay fatherhood tend to combine different paths under one group of “gay fathers” or to concentrate on a single parenthood path (e.g., Shenkman and Shmotkin, 2016; Carone et al., 2017a, b).

While comparisons between different pathways to gay fatherhood are scarce, some comparisons between gay fathers through surrogacy and heterosexual fathers via assisted reproduction have been conducted. Van Rijn-van Gelderen et al. (2018) for example compared the well-being of gay fathers through surrogacy with heterosexual IVF parent families from three European countries (United Kingdom, the Netherlands, and France) and found no differences on parental stress, depression, anxiety, or relationship satisfaction between the two groups.

Shenkman et al. (2018), compared Israeli gay fathers with children from a previous heterosexual relationship and heterosexual fathers and found gay fathers reported higher levels of personal growth (feelings of continued development and self-improvement alongside a sense of personal fulfillment). The authors suggested that gay fathers from a previous heterosexual relationship had probably overcome numerous challenges entailed in the complex course of coming out to oneself and their ex-spouse and children. Coping successfully with such challenges could result in the construction of a new meaning to life, which might then explain the high levels of personal growth displayed. In another Israeli study, gay men who had pursued several different routes to gay fatherhood (fatherhood through surrogacy, a shared parenting agreement with a woman, and adoption) were compared with heterosexual fathers. Some differences between gay and heterosexual fathers emerged showing gay fathers reporting greater satisfaction with life and general happiness than did heterosexual fathers (Erez and Shenkman, 2016). No group differences were observed in self-reported positive and negative emotions. This lack of difference between gay fathers from a variety of routes to parenthood and heterosexual fathers on negative emotions, alongside the absence of differences on levels of neuroticism and depressive symptomatology, was again confirmed by Shenkman and Shmotkin (2019). Thus, in a familistic society such as Israel, success in becoming a gay father might ameliorate the adverse consequences of minority stress, thus resulting in no difference or even more positive psychological well-being outcomes for gay men compared with heterosexual men upon attaining fatherhood (Shenkman and Shmotkin, 2014, 2016, 2019).

Research exploring differences between gay and heterosexual men on Costa and McCrae’s (1992) Big Five personality traits, namely, extraversion, neuroticism, conscientiousness, agreeableness, and openness to experience, has produced mixed results. While some research teams have found no profound differences between the groups on personality traits in countries such Israel and New Zealand (e.g., Greaves et al., 2017; Ifrah et al., 2018), others have indicated that gay men were slightly higher than heterosexual men on agreeableness, conscientiousness, neuroticism, and openness to experience (e.g., Lippa, 2005; Zheng et al., 2011). Significant results have been interpreted in light of minority stress theory (Meyer, 2003) and the possible association between greater exposure to prejudice, discrimination, and social disapproval and particular personality features (Zheng et al., 2011). It was proposed, for example, that gay men and lesbian women may experience, on average, higher levels of neuroticism (e.g., higher levels of anxiety and depression and reduced levels of self-esteem) compared to heterosexual men and women, because of the stress related to their prevalent experience with prejudice and discrimination (Lippa, 2005). However, these studies did not specifically focus upon gay fathers.

### Research Hypotheses

The current research hypotheses were derived from the literature comparing gay fathers (single route and combined routes) with heterosexual fathers and a consideration of the Israeli societal climate that highly esteems childrearing (e.g., Shenkman, 2012; Erez and Shenkman, 2016; Shenkman and Shmotkin, 2019). From the literature on the route to surrogacy for gay men and other results suggesting more positive outcomes in psychological well-being for parents who contended with difficulties prior to parenthood (Taubman-Ben-Ari, 2012, 2014; Shenkman and Shmotkin, 2016), we hypothesized that gay fathers who had become fathers via surrogacy would score higher than heterosexual fathers on parenthood satisfaction and life satisfaction. Considering prior findings suggesting enhanced life meaning for gay fathers through heterosexual relationship in comparison to heterosexual fathers (Shenkman et al., 2018), we hypothesized that the former group would score also higher on life satisfaction.

As research on personality dimensions as a function of fatherhood route is quite novel, and given the exploratory nature of these anticipated analyses, we did not formulate specific hypotheses regarding differences between the study groups on personality dimensions. However, we did expect to find a difference between gay fathers through surrogacy and heterosexual fathers or gay fathers through a previous heterosexual relationship on extraversion, such that gay fathers through surrogacy would report higher levels of extraversion. Our rationale for this centered on the characteristics of extraversion and the characteristics of the surrogacy path for Israeli gay men. The Five Factor Model of personality has situated extraversion as a preference for higher interpersonal interaction, activity level, and stimulation, whereas introversion indicates the opposite tendencies (Costa and McCrae, 1992). Thus, extraverts prefer attending to the outer world of objective events placing an emphasis on active involvement in the environment and in developing larger social support networks whereas introverts do not. We suggest that these features of extraversion are especially relevant for Israeli gay men seeking surrogacy. Because surrogacy is not legal for same-sex couples in Israel, gay men pursuing surrogacy turn to extremely expensive overseas surrogacy services in South-east Asia and the United States of America. This journey to fatherhood therefore requires several active steps to be taken by gay men, such as reaching out to specialist lawyers in Israel and abroad, undergoing specific medical and psychological examinations in Israel and abroad, choosing and securing an egg donor, choosing a surrogate and building a relationship with her, undertaking several journeys to the country of surrogacy while the surrogacy is being conducted, and dealing with the extensive bureaucracy surrounding the registration of a newborn born abroad as an Israeli citizen (Ziv and Freund-Eschar, 2015). Therefore, we thought that the successful pursuit of surrogacy might be associated with extraversion. Similarly, it was suggested that sexual identity disclosure and extraversion might be associated (e.g., Clausell and Roisman, 2009), thus, in the process of pursing surrogacy, which requires multiple disclosures to relevant services, it could be assumed that there would be a link between disclosure, extraversion, and the surrogacy path. Based on these rationales, we predicted that gay fathers through surrogacy would report higher levels of extraversion than heterosexual fathers or gay fathers through a heterosexual relationship.

## Materials and Methods

### Participants

The participants included Israeli gay and heterosexual fathers that were selected from two larger samples. The first, including 692 gay men (aged 16–84, *M* = 42.20, *SD* = 14.23) who were recruited in the years 2010–2016, and the second, including 317 gay and heterosexual men (aged 18–85, *M* = 38.13, *SD* = 9.43) who were recruited in 2013–2014. Participants in both samples were fathers and also non-fathers, and were recruited via targeted sampling (see section “Procedure”). These samples were drawn from a larger research project that explored psychological well-being and adverse experiences among cisgender men throughout their lifespans. Participants for the current analysis who were not biological fathers, who did not become fathers through surrogacy or a heterosexual relationship, and those who identified themselves other than exclusively gay or exclusively heterosexual were excluded from the current analyses as we aimed to focus on the ends of the Kinsey scale. Thus, the sample for the current study comprised: 76 gay men who had become fathers through a heterosexual relationship (mean age 57.84, *SD* = 7.56), 63 gay men who had become fathers through surrogacy (mean age 39.11, *SD* = 5.56), and 78 heterosexual fathers (mean age 38.99, *SD* = 7.90).

Table 1 shows sociodemographic characteristics of the three study groups. Gay fathers through a heterosexual relationship were older than either gay fathers through surrogacy or heterosexual fathers, *F*(2,212) = 169.37, *p* < 0.001, η^2^ = 0.615. Most of the participants in each of the study groups were born in Israel, though gay fathers who had become fathers through a heterosexual relationship were slightly more likely to have been born outside of Israel, χ^2^(2) = 12.06, *p* = 0.002, Cramer’s *V* = 0.235. Most participants had a University level education, and reported an average to high economic status, with gay fathers through a heterosexual relationship reporting a lower economic status than heterosexual fathers and gay fathers through surrogacy, *F*(2,216) = 8.33, *p* < 0.001, η^2^ = 0.072. Most participants reported good or very good physical health, with gay fathers through a heterosexual relationship reporting somewhat poorer health status than heterosexual fathers and gay fathers through surrogacy, *F*(2,214) = 4.20, *p* = 0.016, η^2^ = 0.038. Further, most of the participants were secular, though gay fathers who had become fathers through surrogacy were more likely to declare themselves as secular when compared with gay fathers who had become fathers through a heterosexual relationship and heterosexual fathers, χ^2^(2) = 7.75, *p* = 0.021 Cramer’s *V* = 0.188. Most participants identified as Jewish, and lived in a city. While most gay fathers who had become fathers through surrogacy and heterosexual fathers were in a committed romantic relationship, this was the case for only about a half of the gay fathers who had become fathers through a heterosexual relationship, χ^2^(2) = 20.39, *p* < 0.001, Cramer’s *V* = 0.305. The average number of children was two among heterosexual and gay fathers through surrogacy, and three for the gay fathers through a heterosexual relationship, *F*(2,216) = 26.15, *p* < 0.001, η^2^ = 0.195. The average child’s age was approximately six year for the heterosexual father group, approximately two for gay fathers through surrogacy, and approximately 28 for gay fathers through a heterosexual relationship, *F*(2,215) = 341.98, *p* < 0.001, η^2^ = 0.761. The greatest likelihood of having children living with them at home was among gay fathers through surrogacy, then among heterosexual fathers, and lastly among gay fathers with children from a heterosexual relationship, χ^2^(2) = 128.07, *p* < 0.001, Cramer’s *V* = 0.770. All these significant differences were controlled for in later analyses.

**TABLE 1:** Sociodemographic characteristics of the study groups.

**Variable**	**Heterosexual fathers (*n* = 78)**	**Gay fathers through a heterosexual relationship (*n* = 76)**	**Gay fathers through Surrogacy (*n* = 63)**	**Difference test**
Age (range)	29–66	39–78	30–56	*F*(2,212) = 169.37***, η^2^ = 0.615
*M*	38.99^a^	58.17^b^	39.11^a^	
*SD*	7.90	7.79	5.56	
Place of birth (%)				χ^2^(2) = 12.06**, Cramer’s *V* = 0.235
(0) Israel	92.3	79.5	96.8	
(1) Other	7.7	20.5	3.2	
Education level (%)				*F*(2,216) = 0.46, η^2^ = 0.004
(1) Elementary or no education	1.3	1.3	0	
(2) Partial high school	0	5.1	1.6	
(3) Full high school	5.1	3.8	9.5	
(4) Higher education	5.1	3.8	3.2	
(5) Academic education	88.5	85.9	85.7	
*M*	4.79	4.68	4.73	
*SD*	0.65	0.86	0.70	
Self-rated economic status (%)				*F*(2,216) = 8.33***, η^2^ = 0.072
(1) Low	2.6	1.3	0	
(2) Below average	0	6.4	1.6	
(3) Average	32.1	47.4	22.2	
(4) Above average	46.2	35.9	52.4	
(5) High	19.2	9.0	23.8	
*M*	3.79^a^	3.45^b^	3.98^a^	
*SD*	0.84	0.80	0.72	
Self-rated health (%)				*F*(2,214) = 4.20*, η^2^ = 0.038
(1) Bad	0	0	0	
(2) Not so good	0	2.6	0	
(3) Fair	5.1	16.9	3.2	
(4) Good	46.2	41.6	48.4	
(5) Very good	48.7	39.0	48.4	
*M*	4.44^a^	4.17^b^	4.45^a^	
*SD*	0.59	0.80	0.56	
Self-rated religiousness (%)				χ^2^(2) = 7.75*, Cramer’s *V* = 0.188
(0) Secular	84.6	87.2	98.4	
(1) Other	15.4	12.8	1.6	
Family religion (%)				χ^2^(2) = 1.15, Cramer’s *V* = 0.073
(0) Jewish	98.7	100	98.4	
(1) Other	1.3	0	1.6	
Children at home (%)				χ^2^(2) = 128.07***, Cramer’s *V* = 0.770
(0) No	8.0	78.2	0	
(1) Yes	92.0	21.8	100	
Place of residence (%)				χ^2^(2) = 2.25, Cramer’s *V* = 0.101
(0) City	78.2	84.6	87.3	
(1) Rural	21.8	15.4	12.7	
Relationship status (%)				χ^2^(2) = 20.39***, Cramer’s *V* = 0.305
(0) Not in relationship	19.2	46.2	15.9	
(1) In relationship	80.8	53.8	84.1	
Number of children (range)	1–6	1–8	1–3	*F*(2,216) = 26.15***, η^2^ = 0.195
*M*	1.87^a^	2.79^b^	1.71^a^	
*SD*	0.92	1.25	0.60	
Children’s age^1^ (range)	0.08–31.5	9–47	1–17	*F*(2,215) = 341.98***, η^2^ = 0.761
*M*	5.86^a^	28.29^b^	2.29^c^	
*SD*	7.19	7.95	2.49	

The ANOVA tests regarding age, education level, self-rated economic status, self-rated health, number of children, and children’s age compared the respective mean ratings of the three study groups. Significant pairwise comparisons are noted by different superscripts within each sociodemographic variable (according to Bonferroni *post hoc* tests, *p* < 0.05). ^1^Calculated as the mean age of each participant’s children. * p < 0.05, ** p < 0.01, *** *p* < 0.001.

### Measures

#### Sociodemographic Variables

A 7-point self-rating scale was used to classify participants’ sexual orientation (Kinsey et al., 1948) ranging from 0 (*exclusively heterosexual*, identifying the heterosexual men as participants) to 6 (*exclusively homosexual*, identifying the gay men as participants). We have also assessed self-acceptance of one’s sexual orientation among gay participants by the following item: “To what extent do you accept your sexual orientation?” The item was rated on a scale ranging 1 (*not at all*) to 5 (*very much*). This item is thought to reflect the central component of sexual identity (Elizur and Mintzer, 2001), and a similar item assessment had been used with an Israeli sample previously (Shenkman, 2012). Other sociodemographic queries such as education level (ranging from 1, *elementary or no education* to 5, *University education*), self-rated economic status (ranging from 1, *low*, to 5, *high*), and self-rated religiousness (divided into *secular* versus *traditionalist or religious*), along with their encoded categories, are presented in Table 1. It should be noted that we have used a self-rated economic status measure, i.e., a subjective assessment rather than objective report based on actual income. This subjective measure has proved to be a reliable measure of self-reported economic status with high compliance in previous studies (e.g., Ifrah et al., 2018; Shenkman et al., 2019) and has been shown to be associated with actual income (e.g., Litwin and Sapir, 2009).

#### Big Five Inventory (BFI)

This 44-item measure was designed to allow a quick and efficient assessment of the Big Five personality dimensions: extraversion, neuroticism, conscientiousness, agreeableness, and openness to experience (John and Srivastava, 1999). Each item includes one or two prototypical trait adjectives with some clarifying information. For example, openness to experience is measured by 10 items such as “original, comes up with new ideas” and “curious about many different things.” Respondents are asked to rate the extent to which they are characterized by each item on a scale ranging from 1 (*strongly disagree*) to 5 (*strongly agree*). Each personality dimension was scored as the respondent’s mean of the respective items, where higher scores indicated higher levels of each dimension. Cronbach’s alpha coefficients for extraversion, neuroticism, openness to experience, conscientiousness, and agreeableness in the current sample as a whole were 0.77, 0.86, 0.76, 0.81, and 0.69, respectively. Among heterosexual fathers the coefficients were 0.74, 0.87, 0.64, 0.86, and 0.76. Among gay fathers through a previous heterosexual relationship the coefficients were 0.80, 0.86, 0.75, 0.62, and 0.60. Among gay fathers through surrogacy the coefficients were 0.80, 0.86, 0.86, 0.86, and 0.63. This measure has been widely used worldwide (e.g., Prinzie et al., 2009) including in Israel (e.g., Ifrah et al., 2018).

#### Center for Epidemiologic Studies Depression Scale (CES-D)

Center for Epidemiologic Studies Depression Scale was designed to assess self-reported symptoms associated with depression (Radloff, 1977). This measure consists of 20 items describing major components of depressive symptomology. For each item, respondents were asked to rate how often they had felt or behaved this way in the past week (e.g., “I felt that I could not shake off the blues even with help from my family or friends” and “I felt hopeful about the future,” the latter was one of four reverse-coded items). Ratings ranged from 1 (*rarely or none of the time*) to 4 (*most or all of the time*). The respondent’s score was the items’ mean rating, with higher scores referring to more depressive symptoms. Cronbach’s alpha coefficient in the current sample as a whole was 0.87, and respectively 0.85, 0.87, and 0.89 among the heterosexual fathers, the gay fathers through a previous heterosexual relationship, and the gay fathers through surrogacy. This instrument has been extensively used in research and for clinical purposes (Stansbury et al., 2006) and has been widely used in Israel (e.g., Shenkman, 2012; Shenkman et al., 2017).

#### Satisfaction With Life Scale (SWLS)

This measure was constructed to assess life satisfaction as the cognitive concomitant of subjective well-being (Diener et al., 1985). The measure consists of five items referring to judgments of one’s life (e.g., “The conditions of my life are excellent”) and rated by respondents on a scale ranging from 1 (*strongly disagree*) to 7 (*strongly agree*). The score was the items’ mean rating. Cronbach’s alpha coefficient of SWLS in the current sample as a whole was 0.88, and respectively 0.91, 0.84, and 0.88 among the heterosexual fathers, the gay fathers through a previous heterosexual relationship, and the gay fathers through surrogacy. This measure proved to have highly favorable psychometric properties (Pavot and Diener, 1993) and has been used with Israeli samples (e.g., Shenkman and Shmotkin, 2011).

#### Parenthood Satisfaction

The following item assessed satisfaction from parenthood: “Please rank your satisfaction with being a parent.” This item was rated on a scale ranging 1 (*not at all*) to 10 (*very much*). This item is based on item number 8 from the self-perception of the parental role questionnaire (SPPR; MacPhee and Benson, 1986).

### Procedure

Participants were sampled during one of three waves of recruitment. First, participants were recruited via targeted sampling through various gay social groups across Israel in 2010. Second and third waves of targeted sampling were launched in 2013–2014 and 2015–2016 focusing on recruiting heterosexual fathers and topping up the gay fathers group. By targeted sampling we meant a purposeful, systematic method listing specified sub-populations and aiming to recruit adequate numbers of participants within each of these sub-populations (Watters and Biernacki, 1989). Actual recruitment of participants was then conducted through gay venues, internet forums and websites dealing with LGB issues and/or fatherhood in general, as well as through social media outlets (such as Facebook pages focusing on gay men, gay fathers, or heterosexual fathers) through which contact information for the study was provided to potentially interested participants. The study was advertised to all sub-populations as a study exploring how people maintain happiness in the face of various life adversities. Participants were asked if they were fathers, and if they answered positively, they were further asked to specify the specific route to fatherhood that they had taken (e.g., through adoption, surrogacy, sharing parenting with a woman, or fathering a child through previous heterosexual relationship). All participants were informed that the questionnaires were anonymous and that participation was voluntary, and all participants gave their consent for data entry into the study. Participants were invited to write to the researchers if any question arose, such that a more thorough debriefing could be done. The study was approved for ethical requirement by Tel-Aviv University and the Interdisciplinary Center (IDC), Herzliya, Institutional Review Boards.

### Data Analysis Plan

Data analyses were conducted using SPSS 25. Pearson correlations were first calculated between the main study variables, and preliminary analyses were conducted to identify potential covariates by examining differences between the three groups (gay fathers through surrogacy; gay fathers who had become fathers through a heterosexual relationship; and heterosexual fathers) in the demographic variables using chi-square tests and *F*-tests. Variables with significant differences were controlled in all subsequent analyses.

To test whether the study groups differed on psychological well-being (indicated by depressive symptoms, life satisfaction, and parenthood satisfaction), multivariate analyses of covariance (MANCOVAs) were conducted with pairwise comparisons using Bonferroni-corrected *post hoc* tests. In this analysis, the study group (gay fathers through surrogacy; gay fathers who had become fathers through a heterosexual relationship; and heterosexual fathers) served as the independent variable, depressive symptoms, life satisfaction, and parenthood satisfaction served separately as dependent variables, and nine sociodemographic variables found to significantly differ among the fathers’ groups were used as covariates (age, place of birth, economic status, self-rated health, self-rated religiousness, relationship status, number of children, children mean age, and children residency).

To test whether the study groups differed on the extraversion dimension an analyses of covariance (ANCOVA) was conducted with pairwise comparisons using Bonferroni-corrected *post hoc* tests. In this analysis the study group (gay fathers through surrogacy; gay fathers who had become fathers through a heterosexual relationship; and heterosexual fathers) served as the independent variable, extraversion served as the dependent variable, and nine sociodemographic variables found to significantly differ among the three fathers’ groups were used as covariates. As we did not formulate specific predictions regarding differences between the study groups on the other personality dimensions, four exploratory ANCOVAs were also conducted in the same way with neuroticism, conscientiousness, agreeableness, and openness to experience each serving as dependent variables. Thus, personality dimensions were tested separately by ANCOVAs and not together in a MANCOVA, as they can not be considered as adjacent aspects (a design decision reinforced by the general lack of correlation between most dimensions).

A power analysis using the G^∗^Power 3.1.9.4 computer indicated that a minimum total sample size of 155 people would be needed to detect a medium effect size of η_p_^2^ = 0.06 with a conventional power of 0.80 at 0.05 significance level, using ANCOVA with nine covariates and three groups.

## Results

### Associations Between the Main Variables Under Study

Pearson correlations between the main study variables (the Big Five dimensions, depressive symptoms, life satisfaction and parenthood satisfaction) revealed that higher levels of extraversion were significantly correlated with higher levels of openness to experience, life satisfaction, and lower levels of depressive symptomatology (see Table 2). Neuroticism and depression levels were positively correlated. Higher neuroticism also was correlated with lower levels of conscientiousness, agreeableness, life satisfaction, and parenthood satisfaction. Higher levels of conscientiousness were correlated with lower levels of depressive symptoms and higher levels of life satisfaction. Similarly, higher levels of agreeableness were correlated with lower levels of depressive symptoms and higher levels of life satisfaction. Higher levels of depressive symptomatology were correlated with lower levels of life satisfaction and parenthood satisfaction.

**TABLE 2:** Means, standard deviations and correlations between the study variables.

**Variable**	***M***	***SD***	**1**	**2**	**3**	**4**	**5**	**6**	**7**	**8**
(1) Extraversion	3.52	0.69	–	−0.17*	0.10	0.02	0.16*	−0.33***	0.43***	0.08
(2) Neuroticism	2.44	0.84		–	−0.26***	−0.48***	–0.08	0.50***	−0.35***	−0.23**
(3) Conscientiousness	3.80	0.68			–	0.14	0.11	−0.31***	0.17*	0.03
(4) Agreeableness	3.83	0.57				–	0.09	−0.18*	0.26***	–0.01
(5) Openness	3.98	0.57					–	–0.06	0.09	0.07
(6) Depressive symptoms	1.53	0.39						–	−0.53***	−0.23**
(7) Life satisfaction	5.03	1.25							–	0.30***
(8) Parenthood satisfaction	8.56	1.78								–

*N* = 219. Reported are Pearson correlations. * *p* < 0.05, ** *p* < 0.01, *** *p* < 0.001.

Correlations with the sociodemographic variables that served as controls in our study revealed that being older was significantly correlated with reports of worse physical health (*r* = −0.28, *p* < 0.001), a greater chance of children living outside of home (*r* = −0.74, *p* < 0.001), of having more children (*r* = 0.52, *p* < 0.001), of having older children (*r* = 0.93, *p* < 0.001), and reports of lower levels of satisfaction with parenthood (*r* = −0.17, *p* = 0.011). Higher economic status was significantly correlated with better physical health (*r* = 0.16, *p* = 0.019), greater chance of having children living at home (*r* = 0.20, *p* = 0.004), of having younger children (*r* = −0.18, *p* = 0.007), of being in a romantic relationship (*r* = 0.28, *p* < 0.001), higher levels of life satisfaction (*r* = 0.39, *p* < 0.001), higher levels of parenthood satisfaction (*r* = 0.15, *p* = 0.030), lower levels of depressive symptomatology (*r* = −0.26, *p* < 0.001), and higher levels of extraversion (*r* = 0.18, *p* = 0.009). Better physical health status was significantly correlated with having younger children (*r* = −0.23, *p* = 0.001), a greater likelihood of being in a romantic relationship (*r* = 0.15, *p* = 0.029), and higher levels of life satisfaction (*r* = 0.16, *p* = 0.017) and parenthood satisfaction (*r* = 0.20, *p* = 0.004). Identifying as non-secular (i.e., traditionalist or religious) was significantly correlated with having more children (*r* = 0.20, *p* = 0.003). Having more children was also significantly correlated with having older children (*r* = 0.52, *p* < 0.001), with children not living at home (*r* = −0.41, *p* < 0.001), and lower levels of parenthood satisfaction (*r* = −0.14, *p* = 0.042). Being in a romantic relationship was correlated with higher levels of life satisfaction (*r* = 0.26, *p* < 0.001) and parenthood satisfaction (*r* = 0.27, *p* < 0.001), and lower levels of depressive symptomatology (*r* = −0.23, *p* = 0.001).

### Comparing the Different Pathways to Fatherhood

To test whether gay fathers who had become fathers through surrogacy would score higher than heterosexual fathers on parenthood satisfaction and life satisfaction and whether gay fathers through heterosexual relationship would score higher on life satisfaction in comparison to heterosexual fathers, we conducted a multivariate analysis of covariance (MANCOVA) with *post hoc* pairwise comparisons. Study group (gay fathers through surrogacy; gay fathers who had become fathers through a heterosexual relationship; and heterosexual fathers) served as the independent variable, depressive symptoms, life satisfaction, and parenthood satisfaction served separately as the dependent variable, and the nine sociodemographic variables found to significantly differ between the fathers’ groups (age, place of birth, economic status, self-rated health, self-rated religious classification, relationship status, number of children, mean age of children, and child residency) were used as covariates.

The results indicated a significant multivariate effect, Wilks’s Λ = 0.894, *F*(6,348) = 3.345, *p* = 0.003, η_p_^2^ = 0.055. When looking at the univariate effects (see Table 3), life satisfaction significantly differed among the three groups, *F*(2,176) = 4.827, *p* = 0.009, η_p_^2^ = 0.052. Pairwise comparisons revealed that gay men who became fathers through surrogacy (*M* = 5.31, *SD* = 1.16) scored significantly higher than heterosexual fathers (*M* = 4.70, *SD* = 1.39) on life satisfaction (*p* = 0.002), with no significant differences between gay men who became fathers through surrogacy and gay fathers who became fathers through a heterosexual relationship (*M* = 5.18, *SD* = 1.08; *p* = 0.161) or between gay fathers who became fathers through a heterosexual relationship and heterosexual fathers (*p* = 0.804).

**TABLE 3:** Multivariate Analysis of Covariance of Group (Gay Fathers through surrogacy, Gay Fathers through Heterosexual Relationship, and Heterosexual Fathers) for Psychological Wellbeing Concomitants (Age, Place of Birth, Economic Status, Self-Rated Health, Self-Rated Religiousness, Relationship Status, Number of Children, Children’s Mean Age and Children’s Residency Controlled).

**Dependent measures**	**Wilks’s ∧**	**Descriptives**						***F***	***p***	**Partial eta squared**
		**Gay fathers through surrogacy**		**Gay fathers through heterosexual relationship**		**Heterosexual fathers**				
		***M***	***SD***	***M***	***SD***	***M***	***SD***			
	0.894							*F*(6,348) = 3.345	0.003	0.055
Depressive symptoms		1.50^a^	0.40	1.60^a^	0.43	1.50^a^	0.35	*F*(2,176) = 1.806	0.167	0.020
Life satisfaction		5.31^a^	1.16	5.18^a,b^	1.08	4.70^b^	1.39	*F*(2,176) = 4.827	0.009	0.052
Parenthood satisfaction		9.34^a^	0.90	8.03^a^	2.24	8.27^b^	1.72	*F*(2,176) = 3.556	0.031	0.039

**N* = 188. The MANCOVA test regarding depressive symptoms, life satisfaction and parenthood satisfaction compared the respective mean ratings of the three study groups. Significant pairwise comparisons are noted by different superscripts within each sociodemographic variable (according to Bonferroni *post hoc* tests).*

Univariate effects also showed that parenthood satisfaction significantly differed among the three groups, *F*(2,176) = 3.556, *p* = 0.031, η_p_^2^ = 0.039. Pairwise comparisons revealed that gay men who became fathers through surrogacy (*M* = 9.34, *SD* = 0.90) scored significantly higher than heterosexual fathers (*M* = 8.27, *SD* = 1.72) on parenthood satisfaction (*p* = 0.018), with no significant differences between gay men who became fathers through surrogacy and gay fathers who became fathers through a heterosexual relationship (*M* = 8.03, *SD* = 1.72; *p* = 0.870) or between gay fathers who became fathers through a heterosexual relationship and heterosexual fathers (*p* = 0.216). The differences between gay fathers through surrogacy and heterosexual fathers on life satisfaction and parenthood satisfaction remained significant when Bonferroni corrections were applied.

As shown in Table 3, univariate effects additionally showed that depressive symptomology did not significantly differed among the three groups, *F*(2,176) = 1.806, *p* = 0.167.

To test our prediction that gay fathers through surrogacy would report higher levels of extraversion than either heterosexual fathers or gay fathers through a heterosexual relationship, we conducted univariate analysis of covariance (ANCOVA) with *post hoc* pairwise comparisons. Study group (gay fathers through surrogacy; gay fathers who had become fathers through a heterosexual relationship; and heterosexual fathers) served as the independent variable, extraversion served as the dependent variable, and the nine sociodemographic variables found to significantly differ between the fathers’ groups (age, place of birth, economic status, self-rated health, self-rated religiousness, relationship status, number of children, children mean age, and child residency) were used as covariates.

The results displayed in Table 4 indicated that extraversion significantly differed among the three groups, *F*(2,179) = 4.182, *p* = 0.017, η_p_^2^ = 0.045. Pairwise comparisons revealed that gay men who became fathers through surrogacy (*M* = 3.64, *SD* = 0.72) scored significantly higher on extraversion than heterosexual fathers (*M* = 2.39, *SD* = 0.87; *p* = 0.006). Gay men who became fathers through surrogacy also scored significantly higher on extraversion than gay fathers who became fathers through a heterosexual relationship (*M* = 3.50, *SD* = 0.69; *p* = 0.038). No significant difference was found between gay fathers who became fathers through a heterosexual relationship and heterosexual fathers (*p* = 0.458). The differences between gay fathers through surrogacy and heterosexual fathers on extraversion remained significant when Bonferroni corrections were applied. However, the difference between gay fathers through surrogacy and gay fathers who became fathers through a heterosexual relationship was non-significant.

**TABLE 4:** Analysis of Covariance of Group (Gay Fathers through surrogacy, Gay Fathers through Heterosexual Relationship, and Heterosexual Fathers) for Extraversion, Neuroticism, Conscientiousness, Agreeableness, and Openness (Age, Place of Birth, Economic Status, Self-Rated Health, Self-Rated Religiousness, Relationship Status, Number of Children, Children’s Mean Age and Children’s Residency Controlled).

**Dependent measures**	**Descriptives**						***F***	***p***	**Partial eta squared**
	**Gay fathers through surrogacy**		**Gay fathers through heterosexual relationship**		**Heterosexual fathers**				
	***M***	***SD***	***M***	***SD***	***M***	***SD***			
Extraversion	3.64 ^a^	0.72	3.50 ^b^	0.69	3.40 ^b^	0.63	*F*(2,179) = 4.182	0.017	0.045
Neuroticism	2.55 ^a^	0.80	2.35 ^a^	0.84	2.39 ^a^	0.87	*F*(2,179) = 1.325	0.268	0.015
Conscientiousness	3.73 ^a^	0.76	3.84 ^a^	0.54	3.85 ^a^	0.75	*F*(2,179) = 0.204	0.816	0.002
Agreeableness	3.65 ^a^	0.55	3.99 ^a^	0.48	3.85 ^a^	0.64	*F*(2,179) = 0.795	0.453	0.009
Openness	3.92 ^a^	0.67	3.96 ^a^	0.58	4.03 ^a^	0.47	*F*(2,179) = 1.146	0.320	0.013

**N* = 191. The ANCOVA tests regarding Extraversion, Neuroticism, Conscientiousness, Agreeableness, and Openness compared the respective mean ratings of the three study groups. Significant pairwise comparisons are noted by different superscripts within each sociodemographic variable.*

We also ran four exploratory separate ANCOVAs to examine whether the three fatherhood pathways groups would differ on neuroticism, conscientiousness, agreeableness, or openness to experience. As seen in Table 4, no significant differences among the three groups emerged on either neuroticism, *F*(2,179) = 1.325, *p* = 0.268; conscientiousness, *F*(2,179) = 0.204, *p* = 0.816; agreeableness, *F*(2,179) = 0.795, *p* = 0.453; or openness to experience, *F*(2,179) = 1.146, *p* = 0.320.

Another exploratory ANCOVA was conducted to explore whether gay fathers who became fathers through surrogacy would differ from gay fathers who became fathers through a heterosexual relationship on self-acceptance of one’s sexual orientation, after controlling for the nine sociodemographic covariates. Results indicated that gay fathers through surrogacy did not differ from gay fathers through a previous heterosexual relationship on self-acceptance of sexual orientation, *F*(1,121) = 1.195, *p* = 0.277, partial η^2^ = 0.010 (*M* = 4.80, *SD* = 0.60 and *M* = 4.69, *SD* = 0.52, respectively).

## Discussion

In line with our hypothesis, gay fathers via surrogacy scored higher on parenthood satisfaction and life satisfaction when compared with heterosexual fathers. In line with our prediction regarding the level of extraversion, gay fathers via surrogacy also scored higher on extraversion compared with heterosexual fathers. No significant differences were found between the three fatherhood pathway groups on levels of depressive symptoms or the personality dimensions of neuroticism, conscientiousness, agreeableness, and openness to experience. Contrary to our prediction, gay fathers who became fathers via a heterosexual relationship did not differ from heterosexual fathers on life satisfaction.

The exploratory comparisons between the two studied pathways to gay fatherhood, namely gay fathers through surrogacy and gay fathers through a heterosexual relationship revealed that gay fathers through surrogacy did not differ on any of the psychological well-being indicators from gay fathers who had children through a previous heterosexual relationship. Thus, our research results mostly indicate similarities between the psychological well-being profiles of these two groups.

Our findings revealed greater parenthood satisfaction and general life satisfaction specifically among gay fathers through surrogacy, compared with heterosexual fathers, echo but also extend those of previous studies which suggested that gay fathers within the Israeli context generally indicated higher levels of subjective well-being than did heterosexual fathers (e.g., Erez and Shenkman, 2016). It was suggested that in a society that promotes parenthood as a major marker of social acceptance, yet imposes sociolegal restrictions on access, creates considerable challenge for gay men in their quest for fatherhood (e.g., Shenkman, 2012). Therefore, success in overcoming the difficulties in becoming a parent, may then result in a triumphant sense of personal achievement given the importance of this accomplishment (Armesto, 2002). Personal achievement could be manifested in enhanced well-being (Shenkman and Shmotkin, 2014; Erez and Shenkman, 2016), plus elevated levels of parenthood satisfaction. These findings of elevated parenthood satisfaction and life satisfaction also correspond to findings from studies of heterosexual women with fertility problems who experienced elevated levels of well-being and satisfaction with parenthood upon overcoming obstacles to become a mother (e.g., Taubman-Ben-Ari, 2014). Nevertheless, it could also be argued that gay men with greater well-being and life satisfaction may have more personal resources to pursue parenthood. In particular those with buoyant well-being may have the resilience to undergo the demanding process of surrogacy. Thus, the current differences between gay fathers through surrogacy and heterosexual fathers on life satisfaction may simply reflect these different selection factors. In the same vein self-selection may operate through demographic variables which could in turn differentiate between fatherhood groupings on well-being and on extraversion. Here it should be noted that higher levels of education and income were also shown to associate with higher scores on extraversion (e.g., Viinikainen et al., 2010), and higher extraversion was also found to associate with higher levels of well-being (e.g., Diener et al., 1992). In our study, we aimed to ameliorate some of these issues by controlling multiple sociodemographic variables including economic status and education, when differences between fatherhood groups were found. A longitudinal study could shed further light on this issue.

The lack of difference between gay fathers through surrogacy, gay fathers through a heterosexual relationship, and heterosexual fathers on reported symptoms of depression is in line with those of a previous study showing no differences between gay fathers through surrogacy and heterosexual fathers on parental stress, depression, and anxiety (Van Rijn-van Gelderen et al., 2018). Similarly, the absence of difference between the fatherhood groups on most Big Five personality dimensions has echoed findings from previous studies suggesting no profound differences in general between gay and heterosexual men on personality traits (e.g., Greaves et al., 2017; Ifrah et al., 2018). However, in the current study we did find higher extraversion scores among Israeli gay fathers through surrogacy in comparison to those recorded by heterosexual fathers. This new finding may suggest that the unique pathway to gay fatherhood through surrogacy, could be associated with extraversion as characterized by an active stance when facing the world (Costa and McCrae, 1992), plausibly because the pathway entails very active coping strategies when contacting lawyers, doctors, and surrogates abroad (Ziv and Freund-Eschar, 2015).

Our exploratory comparisons between gay fathers through surrogacy and gay fathers through a previous heterosexual relationship did not detect any well-being differences between these groups, thus we conclude that these two groups are similar in terms of psychological well-being concomitants. This lack of difference is interesting as these two groups can be seen to represent two distinct sociocultural contexts. The gay men who became fathers through surrogacy represented a younger cohort, who grew up mainly in an Israeli society that acknowledged, at list to some extent, gay rights and several options to becoming fathers. In contrast, in our study gay fathers via a heterosexual relationship were representatives of middle aged and older Israeli gay men, who grew up when society proclaimed homosexuality as pathology and neither acknowledged nor offered multiple pathways to gay fatherhood (Morrow, 2001; Tasker and Patterson, 2007). It could be argued that gay fathers through a heterosexual relationship, who risked or endured possibly long lasting stigma, that in turn contributed to maintaining high levels of vigilance and secrecy over their sexual orientation (Kimmel, 2014), might experience adverse well-being outcomes, such as lower life satisfaction (Erdley et al., 2014). Similarly, it could be further argued that the path to gay fatherhood via a previous heterosexual relationship could pose additional difficulties for co-parenting with an ex-partner, that could potentially negatively impact upon life satisfaction and well-being in general (e.g., Tasker, 2013). Nevertheless, our current results indicate that in spite of these potentially very different circumstances, no significant differences between the two groups of gay fathers were found.

Contrary to our prediction, gay fathers via a heterosexual relationship did not report greater life satisfaction than did heterosexual fathers. This was not in line with findings from a previous study that showed that gay fathers with children from a previous heterosexual relationship reported greater meaning in life, as indicated by a sense of personal growth, when compared with heterosexual fathers (Shenkman et al., 2018). It could be argued that personal growth, which is a core indicator for meaning in life, could be considered as a different, sometimes even orthogonal, component of well-being that significantly differs from life satisfaction *per se* (Keyes et al., 2002). Personal growth is representative of eudemonic well-being, namely a reflection upon existential challenges of life in relation to meaning restructuring (Ryff, 1995, 2014). However, life satisfaction is a representative of hedonic well-being, namely a reflection of pleasant and unpleasant affect in sizing up one’s immediate experience (Lucas et al., 1996). Therefore, our findings may extend prior knowledge of Israeli gay fathers through a heterosexual relationship (Shenkman et al., 2018) by locating the difference between this group and heterosexual fathers in the eudemonic sphere, while suggesting no differences on the hedonic one.

### Limitations and Strengths

Several limitations of this study should be noted. First, this study relied on self-reports, thus possibly suffering from biases of self-presentation. Secondly, the study groups were not created from random or representative sampling. Thirdly, the cross-sectional design of the study did not allow inferences about causality. Additionally, parenthood satisfaction was measured through a single item, which poses difficulties with assessing reliability and validity of this measure. It is also unclear whether a small number of participants’ partners completed this survey thus introducing an in-accountable level of dependency within the data. Future research should ensure that this confounding variable is controlled. Finally, while the local viewpoint of the Israeli society may be seen as one of the strengths of this study, it may also entail culture-bound limitations on the generalizability of the results. All these methodological limitations echo prevalent complications in studying gay populations (McCormack, 2014).

Alongside these limitations, the current study has also a number of strengths. First, this was a pioneering examination of differences in psychological well-being between three pathways to parenthood, namely heterosexual fatherhood, gay fatherhood through a heterosexual relationship and gay fatherhood through surrogacy. While previous studies tended to combine several paths to gay fatherhood into one group of gay fathers due to difficulties in reaching a sufficient sample size for each path (e.g., Shenkman and Shmotkin, 2016), the current sample coherently presented the different routes, and avoided confounding effects relating to group compilation (Meyer and Wilson, 2009). Nevertheless, the entry route into heterosexual fatherhood was not explored, although the marital status of the heterosexual fathers was noted. Thus, the use of assisted reproductive technology to achieve fatherhood was not controlled for, and could be a further possible confounding factor in making group comparisons between gay fathers via surrogacy and heterosexual fathers. Second, the study design systematically compared the study groups while controlling for the confounding effects of nine sociodemographic variables, such as the age of the fathers, relationship status, economic status, number of children and children’s age. Another strength of this study lay in the fact that it was conducted in Israel, which presents an interesting sociocultural setting for studies of gay fatherhood by juxtaposing a society that cherishes child rearing and has a fairly liberal legal system, but which also has a traditional religious base enshrining many heterosexist and sometimes homonegative norms into the sociolegal system (Shenkman and Shmotkin, 2011). Findings concerning gay fathers from this society may expand our knowledge of cultural variation in the experiences of gay fathers.

## Conclusion and Implications for Practice

This study was one of the first to compare three routes to fatherhood, namely heterosexual fathers, gay fathers through a heterosexual relationship and gay fathers through surrogacy, on diverse psychological well-being concomitants. Our results mainly emphasize the psychological well-being of fathers and the similarities between the fatherhood groups. Nevertheless, some differences did appear, especially when comparing heterosexual fathers with gay fathers through surrogacy. These differences portray gay fathers through surrogacy as more extraverted and more satisfied with both their parenthood and their life in general. While minority stress theory (Meyer, 2003) usually sheds light on the adversities gay men may endure due to their minority status, the current findings suggest that gay fatherhood, at least within the Israeli context, can be interpreted as a resiliency factor, meaning that in such a familistic and pronatal society, success in becoming a gay father might ameliorate some of the adverse outcomes of minority stress, and therefore result in no differences or even more positive outcomes for gay fathers through surrogacy than for heterosexual fathers on psychological well-being indicators (Shenkman and Shmotkin, 2014, 2019). This interpretation echoes the theoretical framework regarding family systems (Cox and Paley, 1997) that guided our study, and which proposes that psychological outcomes of both children and parents are also influenced by the broader socio-cultural context, and in our current study, the Israeli familistic society.

An application of the current findings appears especially relevant to clinicians working with gay fathers, revealing the potential benefits of fatherhood through surrogacy in regards to psychological well-being. Additionally, it seems that psycho-education focused both on the resiliency as well as the difficulties of gay life trajectories, could allow for a more integrative and perhaps optimistic outlook on gay fathers as a minority group.

Our current results also suggest that the novel comparison of two paths to gay fatherhood, namely through a previous heterosexual relationship or through surrogacy, revealed no differences in psychological well-being even when controlling for sociodemographic factors. Thus, future studies should further explore other variables, such as ones that relates to social and family support, when trying to pinpoint more similarities and differences between these two groups. Future studies should also explore bisexual and transgender fathers who were not included in the current study and are even less studied groups when comparing different routes to fatherhood.

## Data Availability Statement

The datasets generated for this study are available on request to the corresponding author.

## Ethics Statement

All procedures performed in studies involving human participants were in accordance with the ethical standards of the institutional and/or national research committee and with the 1964 Helsinki declaration and its later amendments or comparable ethical standards. The studies involving human participants were reviewed and approved by the Tel Aviv University and the Interdisciplinary Center Herzliya, Institutional Review Boards. The patients/participants provided their written informed consent to participate in this study.

## Author Contributions

All authors listed have made a substantial, direct and intellectual contribution to the work, and approved it for publication.

## Conflict of Interest

The authors declare that the research was conducted in the absence of any commercial or financial relationships that could be construed as a potential conflict of interest.
